# Establishing a 4D-CT lung function related volumetric dose model to reduce radiation pneumonia

**DOI:** 10.1038/s41598-024-63251-0

**Published:** 2024-06-01

**Authors:** Chunmei Liu, Huizhi Liu, Yange Li, Zhiqing Xiao, Yanqiang Wang, Han Guo, Jianmin Luo

**Affiliations:** 1 Department of Radiation Oncology, The Second Hospital of Hebei Medical University, 215 West Heping Road, Shijiazhuang, 050000 Hebei China; 2 Department of Hematology, The Second Hospital of Hebei Medical University, 215 West Heping Road, Shijiazhuang, 050000 Hebei China

**Keywords:** Lung cancer, 4D-CT, Functional imaging, Radiation pneumonia, Medical research, Oncology, Risk factors

## Abstract

In order to study how to use pulmonary functional imaging obtained through 4D-CT fusion for radiotherapy planning, and transform traditional dose volume parameters into functional dose volume parameters, a functional dose volume parameter model that may reduce level 2 and above radiation pneumonia was obtained. 41 pulmonary tumor patients who underwent 4D-CT in our department from 2020 to 2023 were included. MIM Software (MIM 7.0.7; MIM Software Inc., Cleveland, OH, USA) was used to register adjacent phase CT images in the 4D-CT series. The three-dimensional displacement vector of CT pixels was obtained when changing from one respiratory state to another respiratory state, and this three-dimensional vector was quantitatively analyzed. Thus, a color schematic diagram reflecting the degree of changes in lung CT pixels during the breathing process, namely the distribution of ventilation function strength, is obtained. Finally, this diagram is fused with the localization CT image. Select areas with Jacobi > 1.2 as high lung function areas and outline them as fLung. Import the patient's DVH image again, fuse the lung ventilation image with the localization CT image, and obtain the volume of fLung different doses (V60, V55, V50, V45, V40, V35, V30, V25, V20, V15, V10, V5). Analyze the functional dose volume parameters related to the risk of level 2 and above radiation pneumonia using R language and create a predictive model. By using stepwise regression and optimal subset method to screen for independent variables V35, V30, V25, V20, V15, and V10, the prediction formula was obtained as follows: Risk = 0.23656–0.13784 * V35 + 0.37445 * V30-0.38317 * V25 + 0.21341 * V20-0.10209 * V15 + 0.03815 * V10. These six independent variables were analyzed using a column chart, and a calibration curve was drawn using the calibrate function. It was found that the Bias corrected line and the Apparent line were very close to the Ideal line, The consistency between the predicted value and the actual value is very good. By using the ROC function to plot the ROC curve and calculating the area under the curve: 0.8475, 95% CI 0.7237–0.9713, it can also be determined that the accuracy of the model is very high. In addition, we also used Lasso method and random forest method to filter out independent variables with different results, but the calibration curve drawn by the calibration function confirmed poor prediction performance. The function dose volume parameters V35, V30, V25, V20, V15, and V10 obtained through 4D-CT are key factors affecting radiation pneumonia. Establishing a predictive model can provide more accurate lung restriction basis for clinical radiotherapy planning.

## Introduction

Lung cancer is one of the most common clinical malignant tumors in China, and its incidence rate and mortality rate are the first among malignant tumors, posing a serious threat to human health and life. Radiation therapy plays an important role in the treatment of lung cancer, and multiple experiments have shown that the higher the radiation dose, the better the treatment effect. But the higher the dose, the higher the incidence of radiation pneumonia, and the higher the proportion of severe radiation pneumonia. Mild radiation pneumonia requires hospitalization for intravenous therapy, which not only increases the economic burden but also increases the pain of intravenous therapy. And grade V radiation pneumonia is lethal. The current routine prevention method for radiation pneumonia is to control the physical or geometric volume dose evaluation parameters V20 and V30 of both lungs, but this method still has a high incidence of radiation pneumonia. Considering that function varies in different regions of lung tissue and the weight of respiratory function varies, introducing functional factors to evaluate radiation-induced lung injury can more accurately assess the incidence of radiation-induced pneumonia.

At present, lung function measurement instruments are mainly used in clinical practice to measure lung function, with main measurement indicators including total lung volume, residual air volume, vital capacity, and forced expiratory volume in the first second^1,2^. However, this method can only reflect the overall lung function and lacks local detailed information of lung function. Although techniques such as SPECT pulmonary ventilation perfusion imaging^2^, PET13N2, MRI3He, and dual source CT can evaluate local lung function levels, these techniques also have certain limitations, such as poor resolution, high radiation, or high cost. Therefore, a simple and fast new method is needed. In recent years, with the development of imaging technology, four-dimensional computed tomography (4D-CT) technology for chest and abdomen has emerged, which has the advantages of high resolution, short scanning time, low cost, and easy acceptance by patients. It is a promising lung function imaging method. This study explores the extraction of lung function distribution through 4D-CT images and investigates the relationship between volume dose parameters in high lung function areas and radiation pneumonia in radiotherapy plans.

## Methods

**Figure 1:**
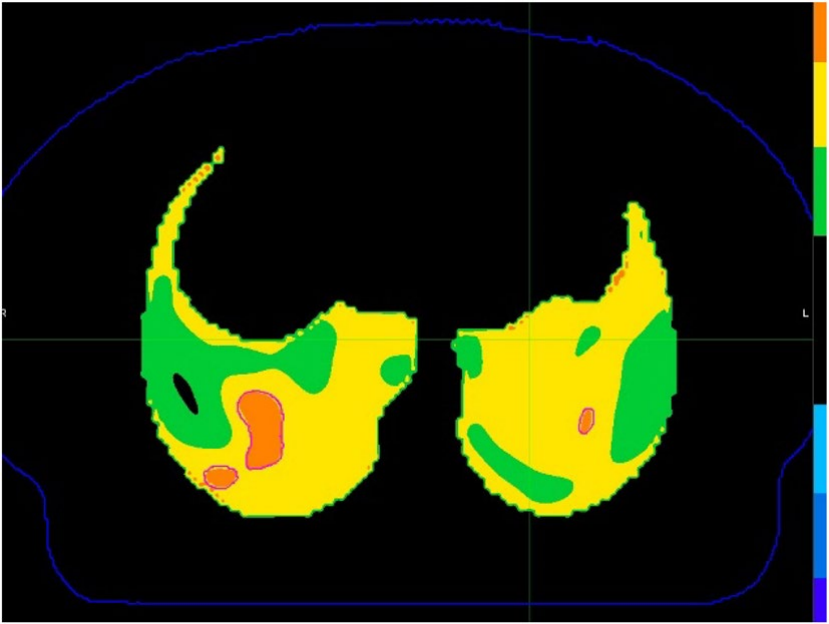
Ventilation function image and the orange area is fLUNG.

**Figure 2:**
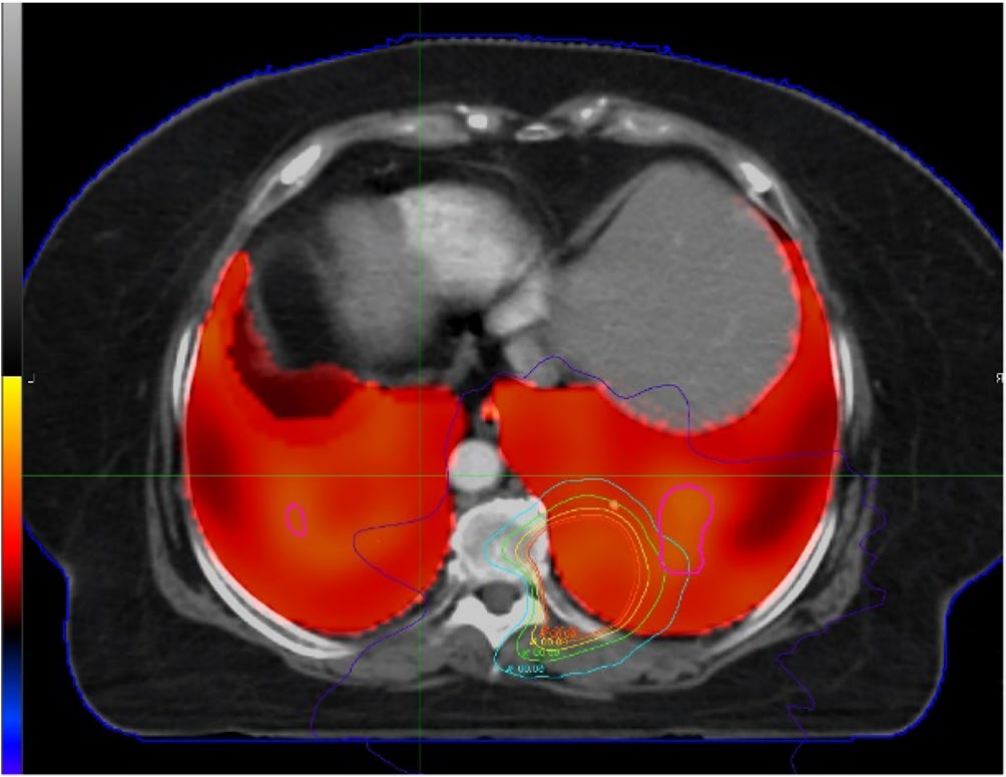
Fusion of lung function image and radiation dose distribution image.

41 pulmonary tumor patients who underwent 4D-CT in our department from 2020 to 2023 were included (Table 1). All 4DCT ventilation images were collected in a supine position. Before scanning, the patient underwent breathing training to ensure smooth and natural breathing. The patient was lying on their back with their forearms crossed in front of their forehead, and was instructed to breathe freely and steadily. The entire lung was scanned in a transverse section using PHILIP 32 row large-aperture spiral CT. The scanning parameters were voltage 120kV, current 100mAs, Cine scan exposure interval 0.45s, layer thickness 3mm, and matrix 512X512. Perform 4DCT scan immediately after completing routine positioning CT scan. 4DCT scanning is performed using the Brilliance Big Bore scanner (Phillips Healthcare, USA) and Varian's real-time position management system (RPM) to track respiratory movements. The scanning parameters are voltage 120 kV, current 300mA, and helix spacing of 3mm. Each bed scans 10 images within a respiratory cycle, and after scanning, reconstructs the four-dimensional images to collect 10 CT images of different time periods during a complete respiratory cycle. The series of images are sent to the MIM workstation, and IGTV is delineated based on the 4D images and positioning images. PIGTV is expanded by 0.5cm, and radiation therapy is given. Regular follow-up is conducted for 1 year at the end of the treatment to obtain information on the patient's development of radiation pneumonia. Then, we retrospectively reviewed the patients who underwent 4D-CT localization and treatment in our department from 2020 to 2023. MIM Software (MIM 7.0.7; MIM Software Inc., Cleveland, OH, USA) was used to register adjacent phase CT images in the 4D-CT series, obtaining a three-dimensional displacement vector of CT pixels when changing from one respiratory state to another. This three-dimensional vector was quantitatively analyzed to obtain a color schematic diagram reflecting the degree of changes in lung CT pixels during the respiratory process, The distribution of ventilation function strength is then fused with the localization CT image. Select areas with Jacobi > 1.2 as high lung function areas and outline them as fLUNG (Fig. 1). Import the patient's DVH image again, fuse the lung ventilation image with the localization CT image, and obtain the volume of fLUNG different doses (V60, V55, V50, V45, V40, V35, V30, V25, V20, V15, V10, V5) (Fig. 2). Analyze the dose volume that affects severe radiation pneumonia using R language.

**Table 1:** The patients’characters.

Characteristic	Radiation pneumonitis(0–1)	Radiation pneumonitis (2–3)	P
Age (median [IQR])	63.00 [54.00, 71.00]	64.00 [55.50, 68.75]	0.989
Vol (median [IQR])	61.91 [29.25, 102.37]	59.44 [24.90, 86.33]	0.769
Sex (%)			0.57
Female	9 (36.0)	5 (31.2)	
Male	16 (64.0)	11 (68.8)	
Segment (%)			0.929
1.7–2.5 Gy/f	11 (44.0)	8 (50.0)	
3 Gy/f	5 (20.0)	3 (18.8)	
5–7 Gy/f	9 (36.0)	5 (31.2)	
Machine (%)			0.707
Accelerator	14 (56.0)	8 (50.0)	
TOMO	11 (44.0)	8 (50.0)	
Location (%)			
Left upper lung	3 (12.0)	2 (12.5)	0.254
Left lower lung	5 (20.0)	4 (25.0)	
Left pulmonary hilum	3 (12.0)	1 (6.2)	
Multiple tumors in the left	0 (0.0)	2 (12.5)	
Right upper lung	4 (16.0)	0 (0.0)	
Right middle lung	1 (4.0)	0 (0.0)	
Right lower lung	5 (20.0)	4 (25.0)	
Right pulmonary hilum	0 (0.0)	2 (12.5)	
Multiple tumors in the right	2 (8.0)	0 (0.0)	
Double lung	2 (8.0)	1 (6.2)

### Ethics declarations and consent to participate

This retrospective study was carried out using the opt-out method for the case series of our hospital. The study was approved by the Research Ethics Committee of the second hospital of Hebei Medical University (approval no.2018-R099) and was conducted in accordance with the 1964 Helsinki Declaration and its later amendments or comparable ethical standards. Informed consent was waived by the Research Ethics Committee of the second hospital of Hebei Medical University due to the retrospective nature of the study and all clinical data was anonymized during analysis to maintain the patient privacy. And all methods were performed in accordance with the relevant guidelines and regulations of the committee of The Second Hospital of Hebei Medical University.

### Statistics

Use R language to screen independent variables using stepwise, Lasso, random forest, and best subset selection methods, draw alignment diagrams/nomograms, and evaluate them using Calibration and ROC curves.

## Results

41 pulmonary tumor patients who underwent 4D-CT in our department from 2020 to 2023 were included. Among them, there are 27 males and 24 females, Treatment was performed using accelerators or TOMO. Three patients underwent double lung radiation therapy (7%), five patients in the left upper lung (12%), nine patients in the left lower lung (22%), four patients in the left lung hilum (9%), two patients in the left lung multiple lesions (5%), five patients in the right upper lung (12%), nine patients in the right lower lung (22%), two patients in the right lung hilum (5%), and two patients in the right lung multiple lesions (5%). Among them, 25 people (61%) did not develop grade 2 or above radiation pneumonia, 14 people (34%) developed grade 2 radiation pneumonia, 2 people (4%) developed grade 3 radiation pneumonia, and no cases of grade 4–5 radiation pneumonia occurred.

### Use R language to screen independent variables through stepwise regression

Select V60, V55, V50, V45, V40, V35, V30, V25, V20, V15, V10, V5 as independent variables, and level 2 and above radiation pneumonia (RP) as dependent variables. Fit the above independent variables and dependent variables using the R language glm function (parameter family = binary), and perform stepwise regression calculation forward and backward using the stepAIC function. Result obtained: Risk = 0.23656–0.13784*V35 + 0.37445*V30-0.38317*V25 + 0.21341*V20-0.10209*V15 + 0.03815*V10.

### Use R language to screen independent variables using Lasso method

**Figure 3:**
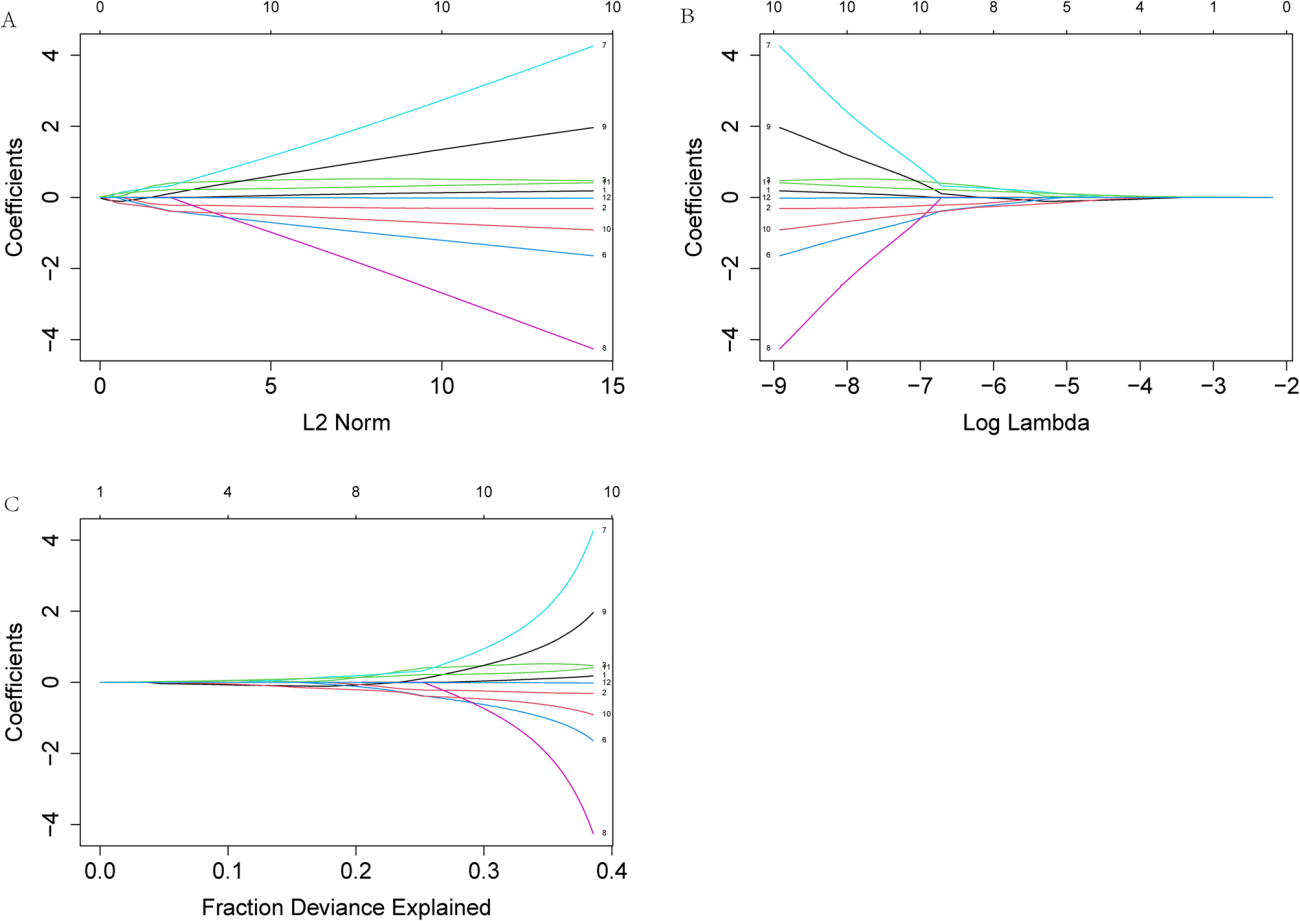
(**A**) The L2 norm and partial regression coefficient graph. (**B**) The lambda and partial regression coefficient graph. (**C**) The explanatory deviation percentage (dev) and partial regression coefficient graph.

Similarly, V60, V55, V50, V45, V40, V35, V30, V25, V20, V15, V10, V5 were selected as independent variables, and radiation pneumonia (RP) of grade 2 and above was selected as the dependent variable. Lasso was performed using the glmnet function in R language. Obtain results: Risk =  − 1.052542 − 1.109038e − 01*V60 + 3.400737e − 02*V50 − 4.149203e − 02*V35 + 1.388844e − 01*V30 − 1.720682e − 01*V15 + 1.125483e − 01*V10 − 5.982490e − 06*V5. And draw Lasso related graphics (as shown in Fig. 3). Figure 3A shows the L2 norm and partial regression coefficient graph. As the L2 norm increases, the absolute value of the partial regression coefficient increases continuously. Figure 3B shows the lambda and partial regression coefficient graph. As lambda increases, the absolute value of the partial regression coefficient decreases continuously. Figure 3C shows the explanatory deviation percentage (dev) and partial regression coefficient graph. As the explanatory deviation percentage increases, the absolute value of the partial regression coefficient increases continuously.

### Use R language to screen independent variables through random forest method

**Figure 4:**
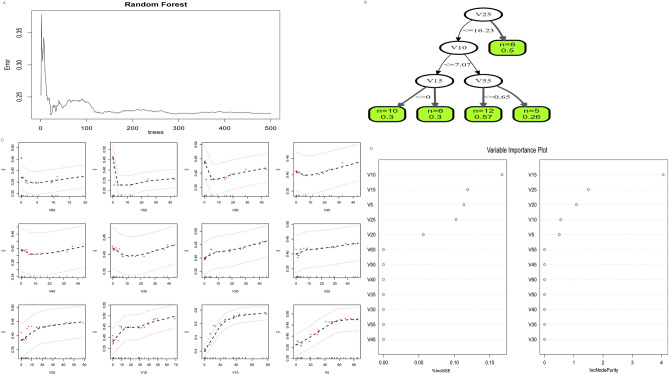
(**A**) The random forest model shows the overall error rate varies with the number of trees, and the number of trees corresponding to the minimum error rate is 1. (**B**) The classification of random forests. (**C**) The differences in each dependent variable. (**D**) The left figure shows the importance of the dependent variable with respect to MeanDecreaseAccuracy, the right figure shows the importance of the dependent variable with respect to MeanDecreaseGini.

Similarly, V60, V55, V50, V45, V40, V35, V30, V25, V20, V15, V10, and V5 were selected as independent variables, and radiation pneumonia (RP) of level 2 and above was selected as the dependent variable. A random forest model was established using the R language randomForest function (Fig. 4A), and the number of trees corresponding to the minimum error rate was determined to be 1. The model was re fitted and the importance score was evaluated using the varImpPlot function (Fig. 4D), Evaluate the MeanDecreaseAccuracy and MeanDecreaseGini indicators. The left figure in Fig. 4D shows the importance of the dependent variable with respect to MeanDecreaseAccuracy, arranged in descending order as V10 > V15 > V5 > V25 > V20 > V60 > V50 > V40 > V35 > V30 > V55 > V45. The right figure in Fig. 4D shows the importance of the dependent variable with respect to MeanDecreaseGini, arranged in descending order as V15 > V25 > V20 > V10 > V5 > V55 > V45 > V60 > V50 > V40 > V30 > V30 > V35 > V35 > V35 > V30. Figure 4C shows the differences in each dependent variable. Apply the rfsrc function again to draw the classification of random forests (Fig. 4B), indicating that the importance of the main influencing factors is ranked as V25 > V10 > V55 > V15.

### Use R language to screen independent variables through the best subset selection method

Similarly, V60, V55, V50, V45, V40, V35, V30, V25, V20, V15, V10, and V5 were selected as independent variables, and radiation pneumonia (RP) of grade 2 and above was selected as the dependent variable. The R language bestglm function was used to screen the independent variables V35, V30, V25, V20, V15, and V10 as the optimal results.

**Figure 5:**
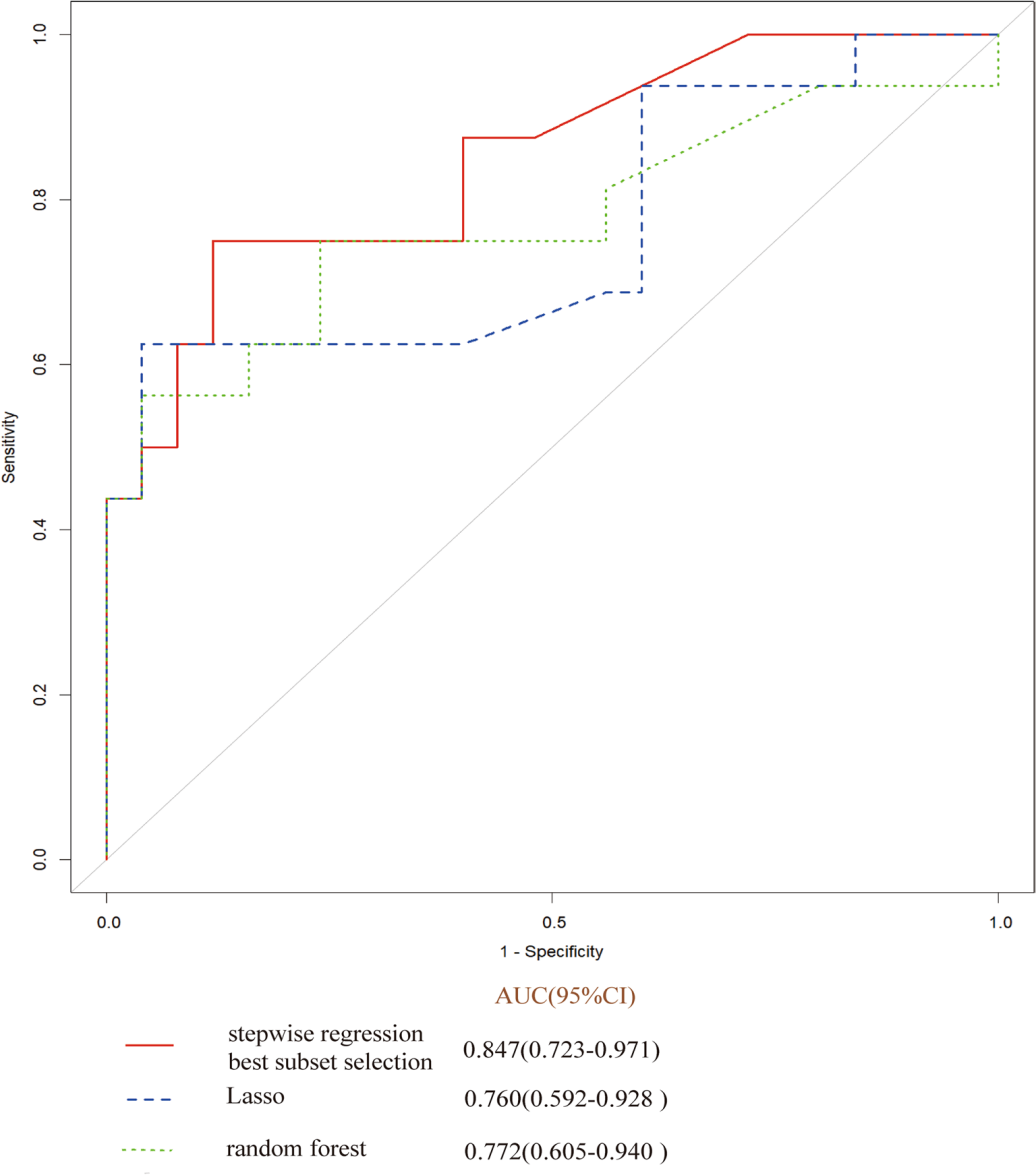
The ROC of various methods.

Analyze the above four methods, including stepwise regression and optimal subset method to screen for independent variables V35, V30, V25, V20, V15, V10, Lasso method to screen for independent variables V60, V50, V35, V30, V15, V10, V5, and random forest method to screen for independent variables V50, V25, V10, and V5. Comparing the ROC of various methods (as shown in the Fig. 5), it can be seen that the stepwise regression and optimal subset method have the highest ROC. Therefore, we selected six variables: V35, V30, V25, V20, V15, V10 to enter the model.

### Using R language for Nomogram analysis

**Figure 6:**
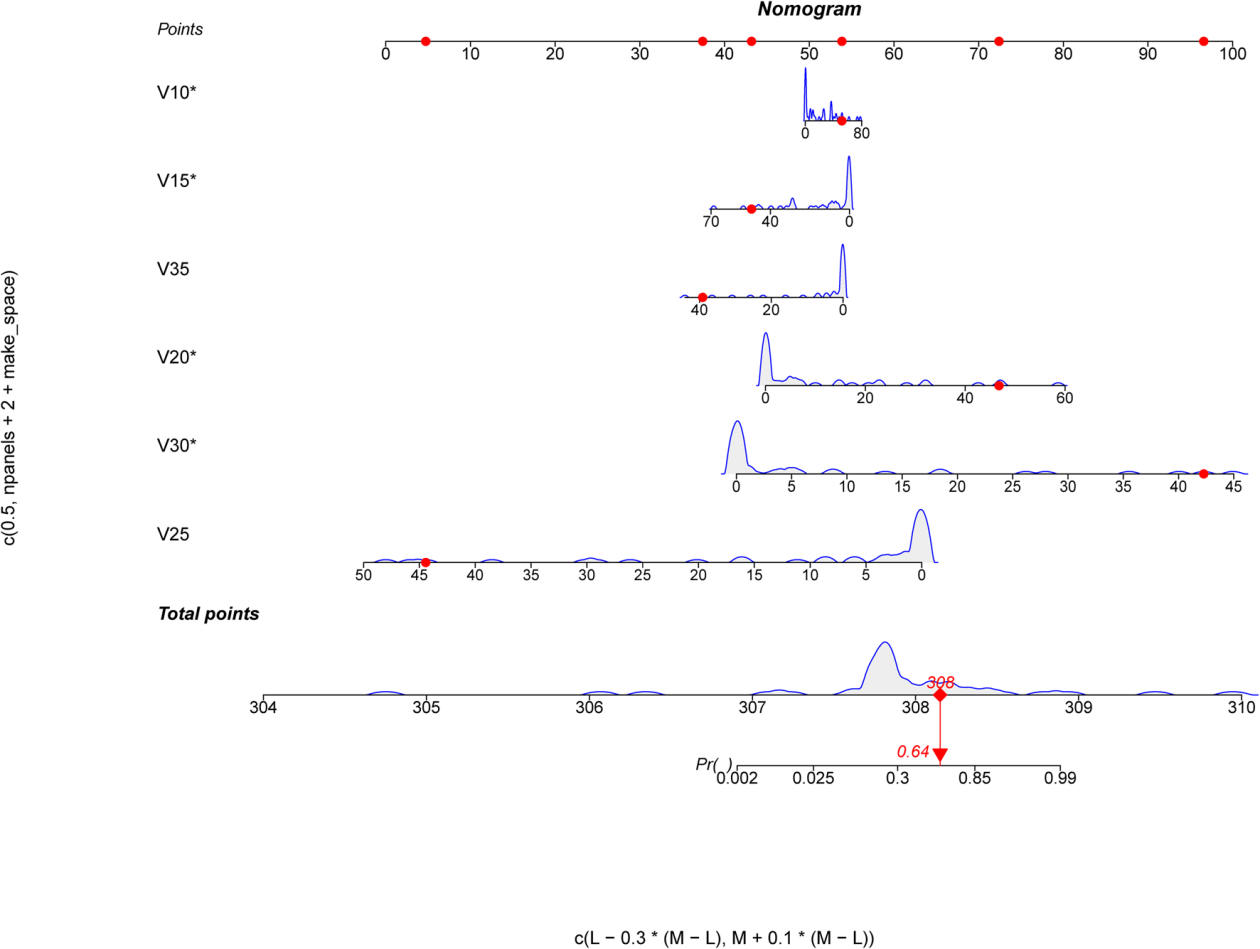
The nomograms of independent variables V35, V30, V25, V20, V15 and V10.

**Figure 7:**
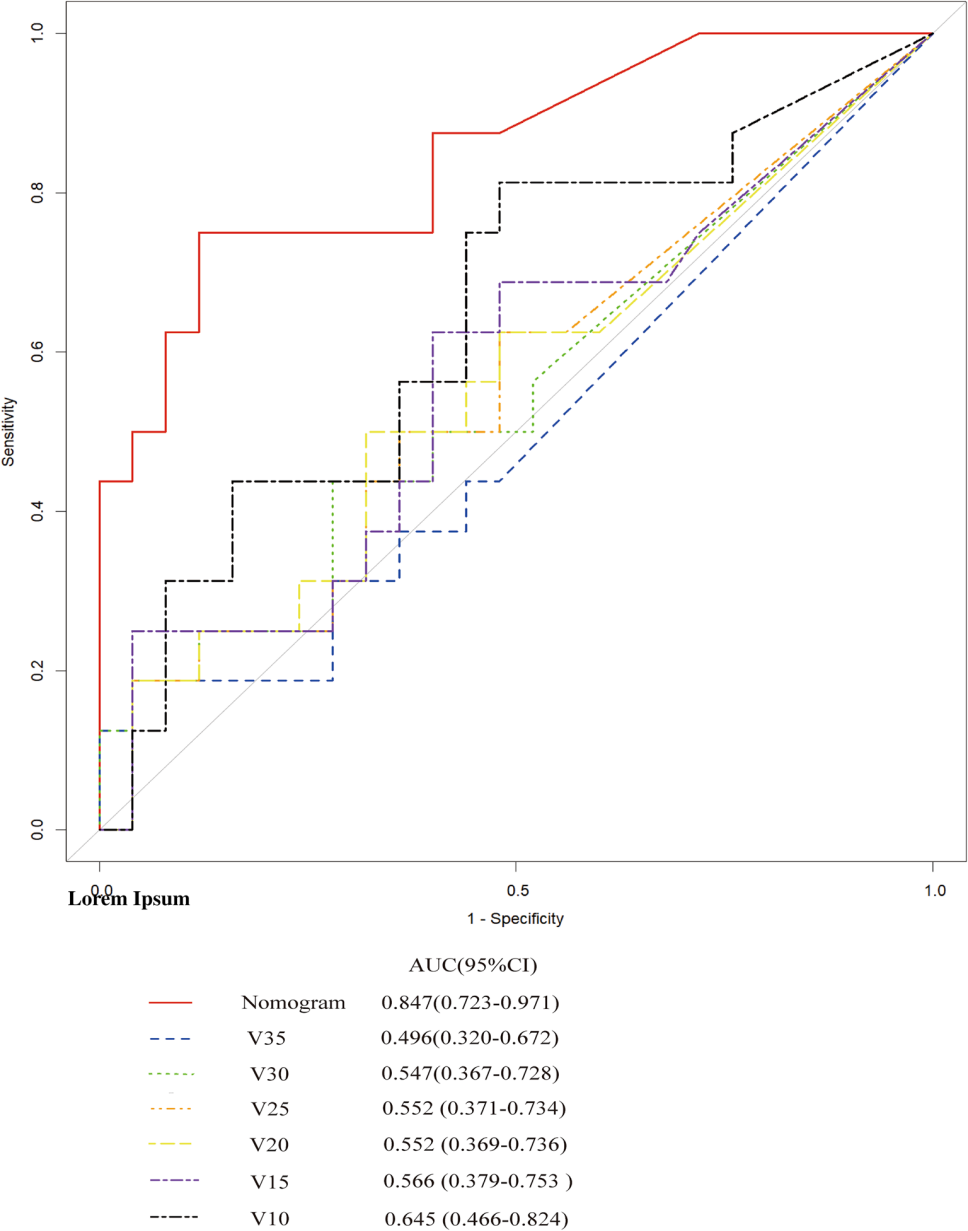
The ROC of the Nomogram model and each variable.

**Figure 8:**
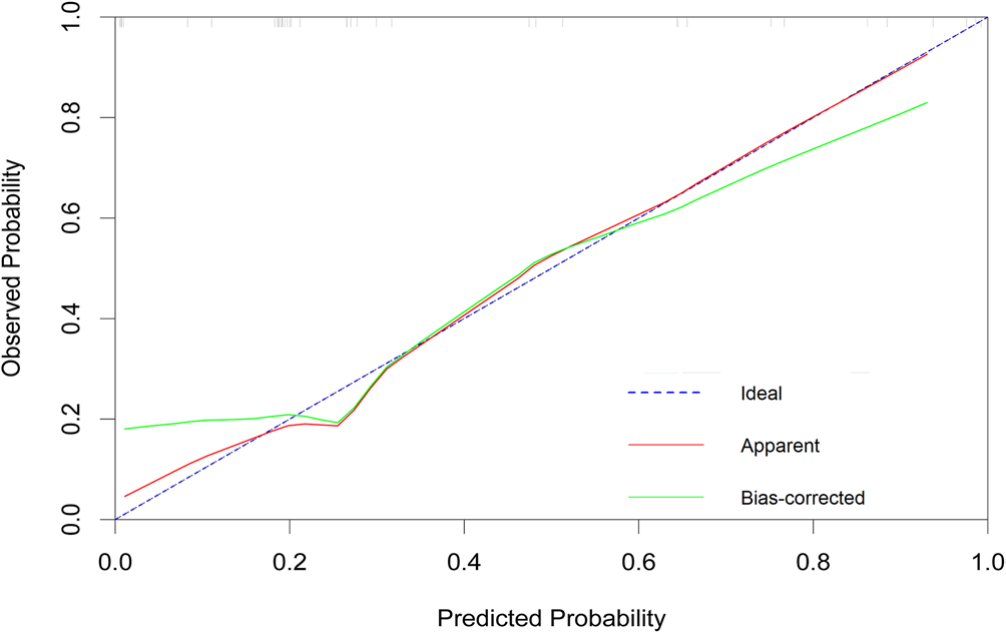
The calibration curve of independent variables V35, V30, v25, V20, V15 and V10.

**Figure 9:**
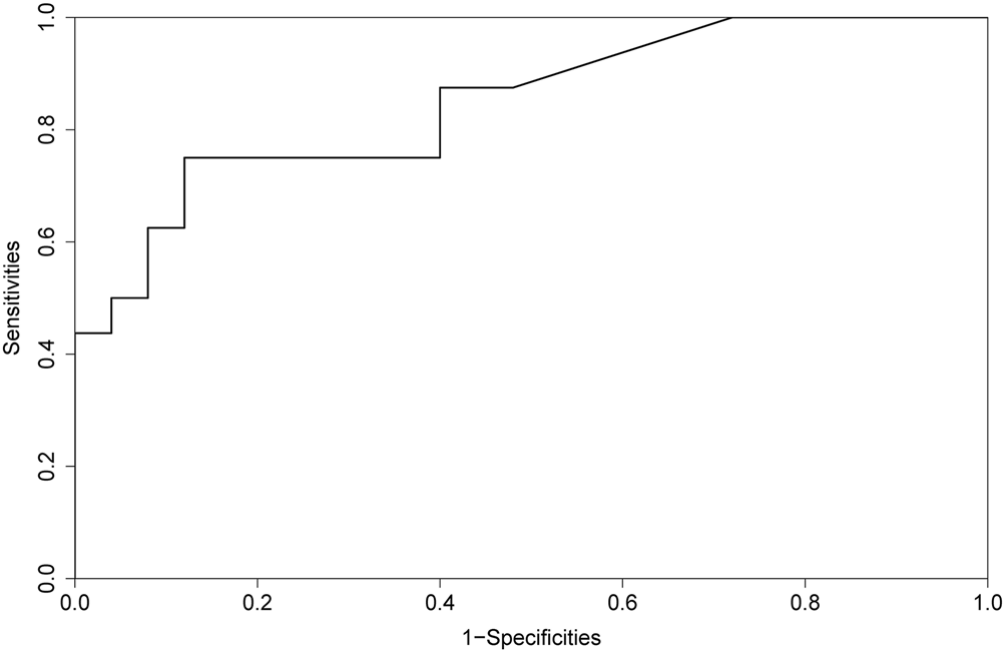
The ROC curve of independent variables V35, V30, V25, V20, V15 and V10.

First, nomogram function of R language is used to analyze the nomograms of independent variables V35, V30, v25, V20, V15 and V10 (as shown in Fig. 6). From the nomograms, it can be seen that the larger V20 and V30 are, the higher the incidence of severe radiation pneumonia is, and V30 has the greatest impact. Then, comparing the ROC of the Nomogram model and each variable (as shown in Fig. 7), it can be seen that the Nomogram model has the best ROC. Then draw the calibration curve through the calibrate function (as shown in Fig. 8). It is found that the bias corrected line and the approximate line are very close to the ideal line, indicating that the predicted value is in good agreement with the actual value. The ROC curve is drawn through the ROC function (Fig. 9), and the area under the curve is calculated: 0.8475, 95% CI 0.7237–0.9713. It can also be determined that the accuracy of the model is very high.

**Figure 10:**
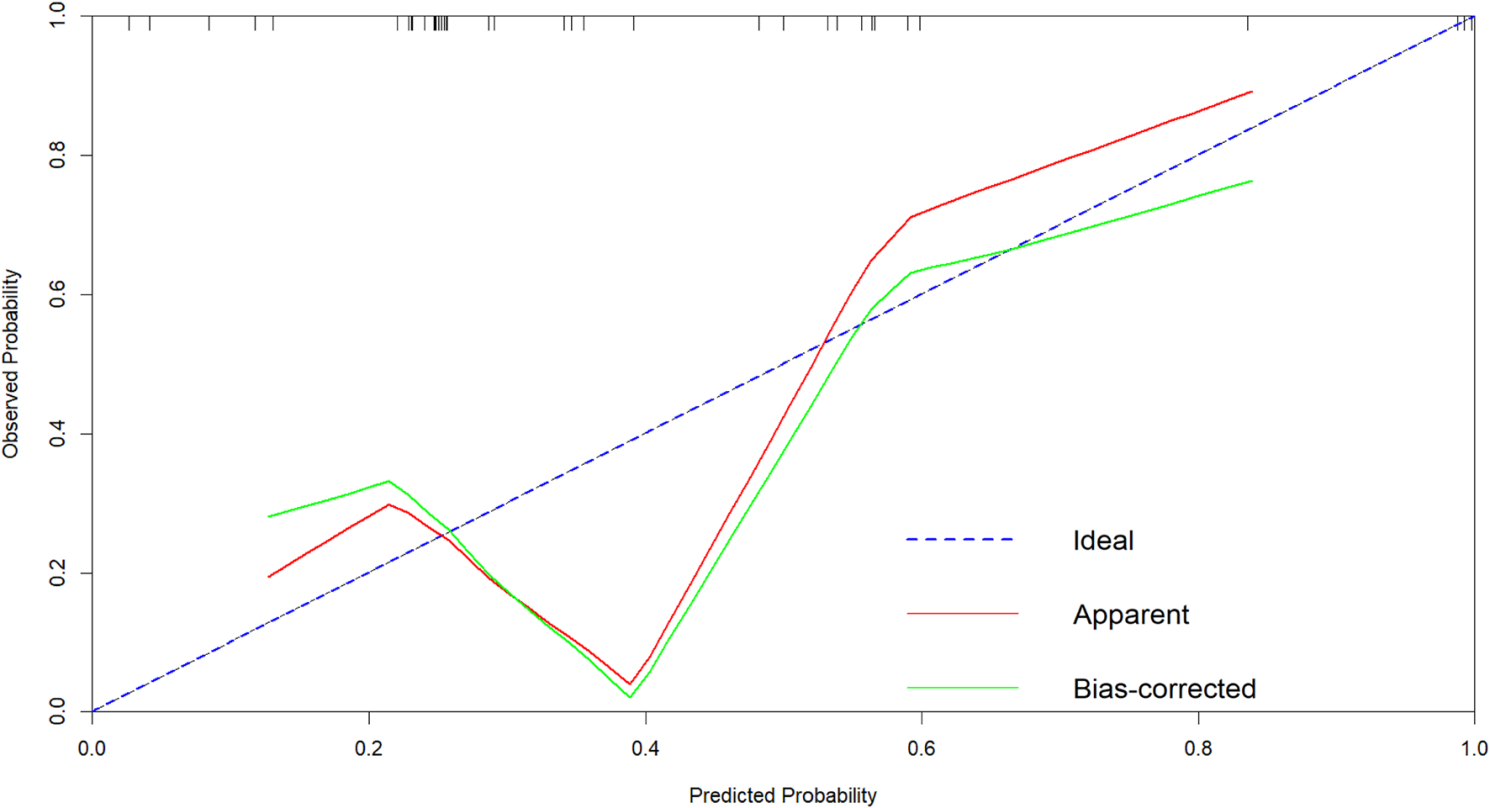
The calibration curve of independent variables V60, V50, V35, V30, V15, V10 and V5.

We continue to analyze the independent variables V60, V50, V35, V30, V15, V10 and V5 by using the nomogram function of R language, and draw the calibration curve through the calibration function (as shown in Fig. 10). It is found that the approximate line is far away from the ideal line, indicating that the consistency between the predicted value and the actual value is not good.

**Figure 11:**
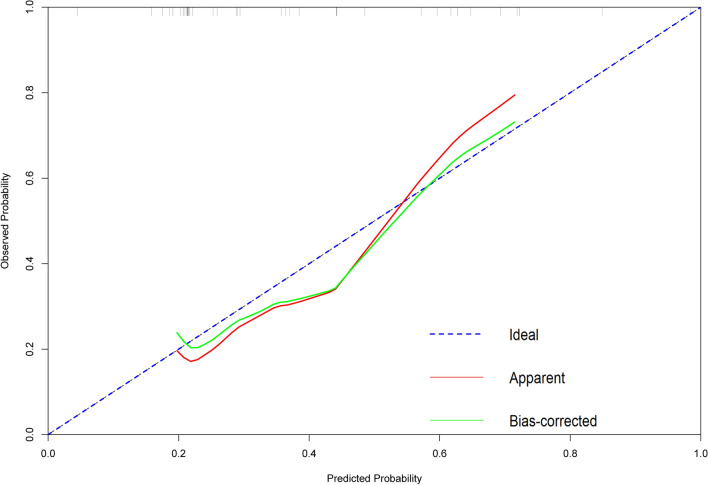
The calibration curve of independent variables V55, v25, V15 and V10.

We continue to analyze the independent variables V55, v25, V15 and V10 by using the nomogram function of R language, and draw the calibration curve through the calibration function (as shown in Fig. 11). It is found that the coincidence between the approximate line and the ideal line is poor, indicating that the consistency between the predicted value and the actual value is not good.

## Discussion

Considering that the blood perfusion in different areas of lung tissue and the weight of respiratory function are different, the introduction of functional factors to assess radiation-induced lung injury can more accurately assess the incidence of radiation-induced pneumonia. At present, the method of preventing severe radiation pneumonitis by thoracic tumor radiotherapy is to control the physical or geometric volume dose evaluation parameters V20 and V30 of both lungs. European Organization for Research and Treatment of Cancer recommends limiting lung V20 to less than 35–37%. However, it also points out that severe radiation pneumonia can occur if V5 is too high, but there is currently no standard recommendation^3^. Zhou's research found that the AUC for predicting the incidence of radiation pneumonia using lung DVH is 0.801–0.804^4^. Hou's research found that the AUC for predicting the incidence of radiation pneumonia using lung DVH is 0.634^5^. Zhang's research found that the AUC for predicting the incidence of radiation pneumonia using lung DVH was 0.637^6^. Puttanawarut's research found that the AUC for predicting the incidence of radiation pneumonia using lung DVH was 0.67^7^. Katsuta's research found that the AUC for predicting the incidence of radiation pneumonia using lung DVH was 0.56^8^. Therefore, the accuracy of predicting radiation pneumonia based on traditional lung DVH is relatively low. We chose to combine lung function to increase the predictive ability of radiation pneumonia. At present, many examinations can provide detailed distribution information of lung function, including proton lung MRI^9^, phase-cycled balanced SSFP (bSSFP) acquisition MRI^10–12^, hyperpolarized gas (HPG) MRI^13^, Electrical impedance tomography (EIT)^14,15^. Fourier decomposition magnetic resonance imaging (FD-MRI)^16–18^, SPECT/CT^2,18–21^, 4D-CT^22–27^, multimodal imaging^28^, and it is applied in radiotherapy planning to avoid higher damage to high-function organs^29–32^. Among these examinations, 4D-CT is the most popular^33,34^, the operation is the simplest, and the radiation needed to be received is low.

Yamamoto performed repeated 4DCT scans on 12 cancer patients, and studied the reproducibility of 4D CT ventilation imaging in two different time periods (15 days (different time periods) and 5 min (the same time period). It was found that the repeatability was poor^23^. Du et al. Quantified the reproducibility of 4D CT ventilation imaging in less than 10 min in three anesthetized mechanically ventilated sheep and nine cancer patients. They found that the reproducibility of sheep was very high, but the reproducibility of patients was relatively poor. Considering that the poor reproducibility was mainly caused by the patients' failure to carry out respiratory training, and all patients in this group had respiratory training for 1–2 days before positioning, so as to ensure that the patients' breathing was stable and natural, Improved repeatability and credibility^35^. Yamamoto analysis showed that the repeatability of 4D CT ventilation imaging may be affected by many factors. One of the important factors is respiratory changes during 4D CT scanning, because respiratory changes can lead to respiratory phase mismatch between CT data segments, which is manifested as artifacts in 4D CT images, and the frequency of such artifacts is very high^22^. Yamamoto found that the repeatability of 4D CT ventilation imaging can be improved by reducing the respiratory rate. Another factor may be the change of the patient's position, including the position of arm elevation. Because the degree of arm elevation is different, it may lead to changes in the shape and anatomical structure of the lung, which may affect the local ventilation of the lung or the image registration between two 4D CT images. This factor can reduce the impact through the use of fixed devices^36^.

Yamamoto studied 12 patients with emphysema. Because the pulmonary function of emphysema area was poor, the 4D-CT ventilation of pulmonary edema area was significantly lower than that of non emphysema area by comparing the low pulmonary function area and emphysema area obtained by 4D-CT^24^. The accuracy of 4D-CT ventilation image was confirmed. Iqbal retrospectively studied 19 patients with locally advanced cancer. He made lung ventilation function images through 4D-CT and found that the plan to protect lung function was completely achievable and would not significantly increase the amount of other organs at risk^26^. Yamamoto's study found that although the plan to protect lung function can reduce function V20 by 5%, it will reduce the fitness of PTV^25,37^. Vinogradskiy included 67 patients with lung cancer. The 4DCT scan of the patients was used to generate the 4DCT ventilation image, which was then used to generate the functional avoidance plan. The plan reduced the dose to the lung function part while delivering the specified tumor dose. The results showed that the incidence of radiation pneumonia ≥ grade 2 was reduced from 25% to 16.4%^34^. Therefore, in this study, the patients were scanned in movie mode in the free breathing state, and the adjacent phase CT images in the 4D-CT series were registered using the workflow of the MIM system. The three-dimensional displacement vector of CT pixels when changing from one breathing state to another was obtained, and the three-dimensional vector was analyzed quantitatively, so as to obtain the color schematic diagram reflecting the change degree of lung CT pixels in the process of breathing, that is, the distribution of ventilation function. Finally, the diagram was fused with the positioning CT image. Select the area with Jacobi > 1.2 as the area with high pulmonary function and sketch it as fLUNG. Then the DVH image of the patient was imported, and the lung ventilation image was fused with the positioning CT image to obtain the volume of different doses of fLUNG (V60, V55, V50, V45, V40, V35, V30, v25, V20, V20, V15, V10, V5). Through R language multiple methods to screen meaningful independent variables, it is found that the model established by stepwise regression method and optimal subset method to screen the independent variables V35, V30, v25, V20, V15, V10 is the most practical, and has passed the calibration curve and ROC curve evaluation. From the nomogram, it can be seen that the larger V20 and V30 are, the higher the incidence of severe radiation pneumonia is, and V30 has the greatest impact. Draw the calibration curve and find that the bias corrected line and the approximate line are very close to the ideal line, indicating that the predicted value is in good agreement with the actual value. Draw the ROC curve and obtain the area under the curve: 0.8475, which also proves that the accuracy of the model is very high. This result is also the first report on the limited conditions of endangering organs and lungs when using lung function images to make radiotherapy plans.

## Conclusion

By calculating V35, V30, v25, V20, V15 and V10 of high-function lung tissue, the prediction of grade 2 and above radiation pneumonia is more accurate. However, there are few cases included in this article, and more cases will be included for verification in the future.

## Data Availability

The datasets generated during and/or analysed during the current study are not publicly available due to privacy of patient information, but are definitely available from the corresponding author if requested. All requests are greeted.
